# EsKiMo II – the Eating study as a KiGGS Module in KiGGS Wave 2

**DOI:** 10.17886/RKI-GBE-2017-107

**Published:** 2017-09-27

**Authors:** Gert B.M. Mensink, Marjolein Haftenberger, Anna-Kristin Brettschneider, Clarissa Lage Barbosa, Hanna Perlitz, Eleni Patelakis, Karoline Heide, Melanie Frank, Franziska Lehmann, Laura Krause, Robin Houben, Hans Butschalowsky, Almut Richter, Panagiotis Kamtsiuris

**Affiliations:** Department of Epidemiology and Health Monitoring, Berlin

**Keywords:** NUTRITION, FOOD CONSUMPTION, NUTRIENT SUPPLY, HEALTH MONITORING, KIGGS

## Abstract

Nutrition plays an important role for health, in particular of children and adolescents. In addition to the baseline German Health Interview and Examination Survey for Children and Adolescents (KiGGS, 2003-2006), the nutrition survey EsKiMo (Eating study as a KiGGS Module) assessed the dietary habits of children and adolescents aged 6 to 17 in detail. In KiGGS Wave 2 (2014-2017) the corresponding module is EsKiMo II. Between June 2015 and September 2017, specially trained nutritionists will visit EsKiMo II participants at their homes. The parents of 6-to 11-year-olds are instructed on how to complete food records on four randomly chosen days - three consecutive days, followed later by an additional day. Participants aged 12 to 17 are interviewed personally on their food intake during the past four weeks with the dietary interview programme DISHES. Further information, for example, regarding dietary supplements is also recorded. EsKiMo II will provide an up-to-date and representative overview of the current nutrition status of 6-to 17-year-olds living in Germany, and it allows analysing changes in dietary behaviour over time. EsKiMo II can identify shortcomings in the nutrition of children and adolescents and thus may contribute with important information to nutrition and health policy.

## 1. Background and objective

Eating and drinking are essential for our life, and individual dietary habits have great influence on our physical and mental health. An adequate diet is particularly important for the growth and health development of children and adolescents. Compared to adults, children require a higher amount of nutrients per kilogramme of body weight. Due to their lower body weight and an immune system which is still developing over the first years of their life, children constitute a particularly vulnerable group for the health implications of food contaminated with pathogens or other harmful substances. Additionally, dietary habits generally develop during childhood and have implications for people’s dietary behaviour at adult age [1]. Monitoring potential health risks related to food intake and improving dietary habits are important tasks of nutrition and health policy. Keeping track of population dietary behaviour on a regular basis is therefore necessary.

In the context of the German Health Interview and Examination Survey for Children and Adolescents (KiGGS) of the Robert Koch Institute (RKI), a food frequency questionnaire is used for participants aged 3 and older to obtain both the frequency and the respective portion size of certain food groups that were consumed during the past four weeks [2, 3]. While this information does provide an impression of respondents’ regular dietary behaviour, it cannot, however, answer more complex questions, for example to identify deficits in respondents’ nutrient supply.

**EsKiMo II**

Second Wave of the Eating study as a KiGGS Module, 2015-2017

**Acronym:** EsKiMo - Eating study as a KiGGS Module

**Implementation:** Robert Koch Institute

**Aim:** Providing an up-to-date representative overview of the dietary habits of children and adolescents aged 6 to 17 in Germany.

**Study design:** Cross-sectional study based on a modified diet history interview and food records

**Population:** Children and adolescents with permanent residence in Germany

**Sampling:** EsKiMo II participants are randomly selected from the cross-sectional sample of KiGGS Wave 2 (registry office sample). Being invited to EsKiMo II requires participation in KiGGS Wave 2.

**Age range:** 6 to 17 years

**Sample size:** at least 2,400 participants

**Survey period:** June 2015 - September 2017

More information in German is available at www.rki.de/eskimo

This led, in the context of the RKI’s KiGGS baseline study (2003-2006), to the implementation of EsKiMo (Eating study as a KiGGS Module, referred to as EsKiMo I in the following), the first representative survey of the dietary behaviour of children and adolescents aged 6 to 17 in Germany [4, 5]. Funding was provided by the Federal Ministry for Consumer Protection, Food and Agriculture, which today is the Federal Ministry of Food and Agriculture (BMEL). Given that data collection for EsKiMo I took place ten years ago, KiGGS Wave 2 (2014-2017) will include the EsKiMo II module, funded by the BMEL. The Federal Institute for Risk Assessment (BfR) will conduct in addition to EsKiMo II a further module on nutrition (KiESEL – the children’s nutrition survey module in KiGGS Wave 2), assessing the dietary habits of children below six years [6].

As a module of the KiGGS study, EsKiMo II will provide the basis for differentiated analyses for example of the relation between dietary habits, socio-demographic criteria (such as size of town, social status, and education), behavioural factors (such as levels of physical activity, use of media or smoking), as well as a diverse set of health parameters (biochemical and physiological measurements) and diseases. Some of the planned analyses will require data collection on food intake to take place as soon as possible after data collection for KiGGS Wave 2, since certain parameters (such as blood values) may be subject to change over time. Data for EsKiMo II will be collected between June 2015 and September 2017. EsKiMo II aims to provide an up-to-date overview of the dietary habits of children and adolescents aged 6 to 17 living in Germany.

## 2. Methodology

### 2.1 Study design and sampling

**Figure 1:**
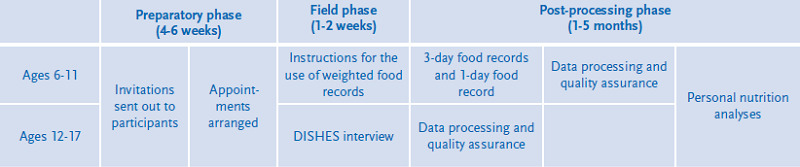
Organisational process of EsKiMo II field phases Own figure

**Figure 2:**
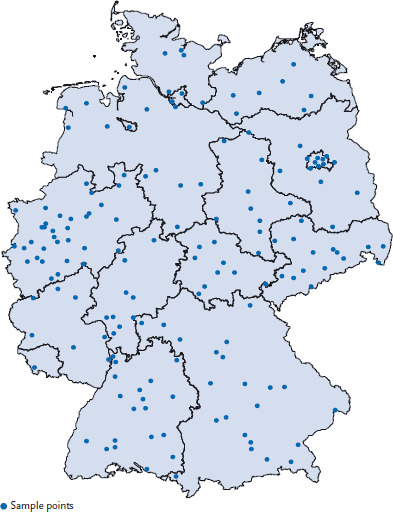
Sample points of EsKiMo II Source: RKI

Participants of EsKiMo II are sampled from the cross-sectional study population of KiGGS Wave 2 (aged 6 to 17), mostly those who took also part in the physical examination of KiGGS Wave 2, and partly those who only answered the KiGGS questionnaire. The target population and sampling for KiGGS Wave 2 is described in detail in the article New data for action. Data collection for KiGGS Wave 2 has been completed in this issue of the Journal of Health Monitoring. Participants receive a written invitation to EsKiMo II three to six months after taking part in KiGGS Wave 2 and about six weeks prior to the scheduled date of the nutrition survey in their locality (routes). Participation and appointments for EsKiMo II are arranged by telephone (Figure 1). During a route, participants are visited at their homes in parallel at several KiGGS Wave 2 sample points (Figure 2) by specially trained nutritionists. Like in KiGGS Wave 2, the order of routes ensures a broad distribution of the regions visited across Germany within seasons, to account for seasonal differences. The survey aims for a net sample of at least 2,400 children and adolescents.

EsKiMo II received an approval from the ethics committee of the Hannover School of Medicine (number 2275-2015). Germany’s Federal Commissioner for Data Protection and Freedom of Information has been informed about and has also approved the survey. Parents and guardians of all participants as well as adolescents aged 14 and older have provided their informed consent to participate in the survey.

### 2.2 Assessment methods and testing instruments

**Figure 3:**
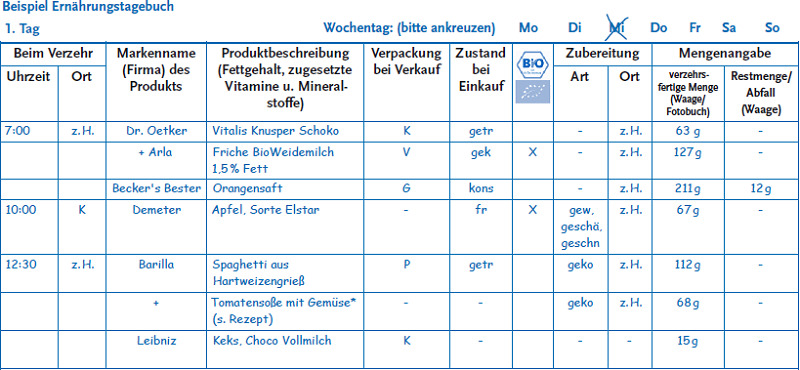
Sample food record page (in German) Source: RKI

As in EsKiMo I, different assessment instruments are used for the specific age groups. For children aged 6 to 11, the parents (or guardians) are asked to use weighted food records to record children’s food intake on three consecutive days, followed by an additional 1-day weighted food record at a later point in time. A randomisation process is used to determine the recording days. The minimum timespan between the 3-day-weighted food record and the 1-day-weighted food record should be two weeks, the maximum timespan three months. The parents (or guardians) are instructed on how to record their children’s food intake. Entries are to include an exact description of the foods consumed as well as information on the brand, product name, fat content (for example of cheese), the actual amount of food on the plate, and leftovers (Figure 3). Details on the time of consumption, place of food preparation/consumption, the state when purchased (for example raw), on how the meal was prepared, and what type of packaging it came in (for example plastic) are to be recorded as well. In addition, participants are asked to provide the recipes of self-prepared meals. Participants are given kitchen scales and instructions on how to use them, as well as a picture book showing portion sizes. A simplified version of the food record that children can fill out themselves is provided for situations where participants cannot weigh meals (for example in school canteens and/or restaurants). The simplified version of the food record requires participants to describe meals as precisely as possible. Parents are asked to discuss the entries with their children at home. The amounts consumed are estimated based on the picture book or household measures. The picture book aims to improve the accuracy of participants’ estimates. It was adapted for EsKiMo II and contains pictures provided by the International Agency for Research on Cancer (IARC) and the Pilot study for the Assessment of Nutrient intake and food Consumption Among Kids in Europe (PANCAKE) [7, 8]. Parents (or guardians) are also asked to weigh and record in advance the food and beverages that children take with them to school, as well as any leftovers. For children who have school meals, parents (or guardians) are asked to provide the menu where possible.

Telephone support is available to respond to any possible question of participants. Participants return the completed food record to the RKI via a post-paid envelope. On the scheduled date they receive the 1-day-weighted food record, which is also to be sent back after completion. After completion, survey participants receive a personal nutrition analysis and a voucher. The information provided in the food records is processed with version 5.3 of the EAT software (Paderborn University) using the codes of the German Nutrient Database version 3.02 [9]. If necessary, discrepancies or missing information is clarified by telephone.

**KiGGS Wave 2**

Second follow-up to the German Health Interview and Examination Survey for Children and Adolescents

**Data owner:** Robert Koch Institute

**Aim:** Providing reliable information on health status, health-related behaviour, living conditions, protective and risk factors, and health care among children, adolescents and young adults living in Germany, with the possibility of trend and longitudinal analyses.

**Study design**: Combined cross-sectional and cohort study conducted as an examination and interview survey

**KiGGS cross-sectional study**

**Population:** Children and adolescents with permanent residence in Germany

**Sampling:** Samples from official residency registries - randomly selected children and adolescents from the 167 cities and municipalities covered by the KiGGS baseline study

**Age range:** 0-17 years

**Sample size:** Approximately 15,000 participants

**KiGGS cohort study**

**Sampling:** Re-invitation of everyone who took part in the KiGGS baseline study (2003-2006; aged between 0 and 17 at that time) and who was willing to participate in a follow-up

**Age range:** 10-29 years

**Sample size:** Approximately 10,000 follow-up participants

**Survey period:** September 2014-August 2017

**Modules:** BELLA, EsKiMo, GerES, KiESEL, MoMo

More information is available at www.kiggs-studie.de/english

**Figure 4:**
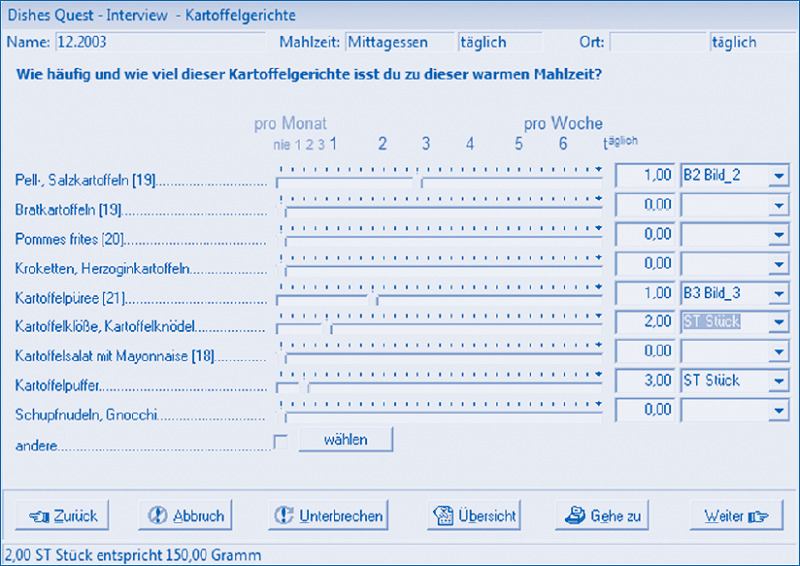
Example of a DISHES interview mask (in German) Source: RKI

With participants aged 12 to 17 a personal dietary interview is conducted during the home visit using the DISHES software (Dietary Interview Software for Health Examination Studies). Developed at the RKI, DISHES is a tool to record regular dietary habits based on a modified diet history method. This method documents the frequency and portion size of meals during the past four weeks (Figure 4), the collected data are internally coded according to the German Nutrient Database version 3.02 [9]. Portion sizes are estimated using tableware and the picture book mentioned above. The instrument has been validated for adults [10]. Provided respondents give their consent, DISHES interviews are recorded digitally to allow staff to clarify cases where the data provided by participants does not seem plausible. In exchange, adolescents are remunerated and receive a personal nutrition analysis.

For both age groups, the survey also records details on school meals, diets, consumption of dietary supplements, as well as information including height and weight. This final item is important, because height and weight may be subject to change in the interval between data collection of KiGGS Wave 2 and EsKiMo II. EsKiMo II also includes participants who were not previously examined in the context of KiGGS Wave 2. The participants’ current weight is required to evaluate their diet.

## 3. Discussion and outlook

EsKiMo II will provide again up-to-date representative data for Germany on the dietary behaviour and nutrient intake of children and adolescents aged 6 to 17. This overview is complemented by data on even younger children collected in the KiESEL survey. This means that a comprehensive data set on the dietary behaviour of children and adolescents of all ages will be available. For nutrition research, food and health policy, as well as for the implementation and evaluation of prevention measures, this data constitutes an important source of information. Besides the Federal Ministry of Food and Agriculture (BMEL) and the Robert Koch Institute, further ministries including the Federal Ministry of Health (BMG), the Federal Ministry for the Environment, Nature Conservation, Building and Nuclear Safety (BMUB), and subordinated institutes such as the Federal Institute for Risk Assessment (BfR), the German Environment Agency (UBA), as well as the European Food Safety Authority (EFSA) are greatly interested in up-to-date data on the dietary behaviour of children and adolescents in Germany. This is because this data helps to identify deficits in the nutrition situation and to develop corresponding consumer protection measures.

Because food supply and therefore also dietary behaviour are changing constantly, surveys on dietary behaviour should be conducted at regular intervals. During the past years, for example, the number of gluten-free, vegetarian, and vegan products on offer has increased significantly. The contents of products such as breakfast cereals are also regularly modified. Consumption of exotic products in Germany has increased as a result of the globalisation of trade. This steadily expanding and changing supply of foods increases the difficulties of recording data on food consumption. Therefore, within EsKiMo II, food composition information on foods not yet included in the German Nutrient Database as well as dietary supplements is continually gathered.

The combination of EsKiMo I (2006) and EsKiMo II (2015-2017) for the first time enables a comprehensive analysis of changes in the dietary habits of children and adolescents in Germany over the past ten years. This is facilitated by the largely identical design and methods used in both surveys. The most important differences between both surveys are that in EsKiMo II data collection was one year longer and that food amounts are weighed instead of estimated. Both of these aspects ought to be considered when interpreting the results. To improve diet-related risk assessment, EsKiMo II uses a 3-day-weighted food record and an independent 1-day food record. EsKiMo is therefore now in line with the standards of other institutions such as EFSA [11]. All of the instruments used in EsKiMo II were developed in close collaboration with the KiESEL study team, a step that will ensure a high degree of comparability between both nutrition surveys.

The food record method produces a detailed and complete appraisal of consumed foods. Frequently, however, food consumption changes while conducting a record. This method is used for young children, in particular, because they cannot be interviewed on their dietary behaviour yet [12]. With adolescents, the willingness to keep such a diary for three consecutive days is probably significantly lower than among parents of younger children. Moreover, adolescents in particular spend a lot of time outside of their homes, which could make filling out a food record difficult. This is why for this group the DISHES interview was used.

Home visits will be concluded in September 2017. A more detailled description of the study design and methods is available elsewhere [13]. First results for EsKiMo II will be available in 2018. EsKiMo II results will be made available to policy-makers, science, and the interested public. Corresponding publication formats are being considered, such as project reports, press releases, and German and English language publications in academic journals.

## Key statements

- Adequate nutrition is essential in ensuring the healthy development of children and adolescents.
- EsKiMo II provides an up-to-date overview of the dietary behaviour of 6-to 17-year-olds in Germany.
- The data provide the basis for comprehensive analyses of trends in dietary habits over time of 6-to 17-year-olds.
- EsKiMo II is an important source of information for nutrition and health policy.
